# Pre- and Post-treatment Changes in Systemic Inflammatory Biomarkers After Oral Appliance Therapy for Obstructive Sleep Apnea: A Systematic Review and Meta-Analysis

**DOI:** 10.7759/cureus.113045

**Published:** 2026-07-20

**Authors:** Arwa Hussain Chaiwala, Hussain Chaiwala, Sachin Franklin, Lussi Y Ronquillo Aguilar, Shweta Bhardwaj, Samantha Rickman

**Affiliations:** 1 Family and Community Medicine, Southern Illinois University School of Medicine, Springfield, USA; 2 Implantology, New York University (NYU) College of Dentistry, New York, USA; 3 Dentistry, Central Counties Health Center, Springfield, USA; 4 Dentistry, Eastern Dental of Parsippany, Morris Plains, USA; 5 Dentistry, Indiana University School of Dentistry, Indianapolis, USA

**Keywords:** obstructive sleep apnea, oral appliance, oral applicance therapy, systemic inflammation, systemic inflammatory markers

## Abstract

Systemic inflammation often accompanies obstructive sleep apnea (OSA), along with higher chances of heart-related metabolic issues. Although oral appliances serve as an accepted option instead of continuous positive airway pressure (CPAP), their influence on markers of systemic inflammation lacks clear evidence. The aim of this systematic review and meta-analysis was to assess changes in systemic inflammation markers following oral appliance therapy (OAT) among individuals diagnosed with OSA. Following Preferred Reporting Items for Systematic Reviews and Meta-Analyses (PRISMA) standards, a systematic review and meta-analysis was conducted. Databases such as PubMed, Embase, Scopus, Web of Science, and Cochrane Library were examined. Studies assessing inflammatory markers before and after therapy among adults with OSA undergoing OAT formed part of the selection. For pooling results, reliance was made upon a random-effects framework. The present research was registered in PROSPERO with the ID CRD420261340212. From a total of 1,974 records screened, six studies qualified for inclusion in the qualitative analysis; among them, two contributed data for each outcome assessed through meta-analysis. Despite pooling outcomes across trials, no meaningful changes emerged in tumor necrosis factor-alpha levels (p = 0.536), nor in C-reactive protein (p = 0.061), or interleukin-6 concentrations (p = 0.230). Consistency between study findings showed minimal variation (I² = 0%). Despite improved clinical results in OSA, OAT shows minor impact on markers of systemic inflammation. To confirm these findings, broader trials with rigorous and uniform designs are needed.

## Introduction and background

Obstructive sleep apnea (OSA), affecting millions of people worldwide, is one of the most commonly encountered sleep-related breathing disorders characterized by frequent episodes of upper airway collapse during sleep, leading to intermittent hypoxia, sleep fragmentation, and systemic complications [1]. Literature has revealed that OSA is not only an airway disorder but also a chronic inflammatory condition [2,3]. Systemic markers like C-reactive protein (CRP), interleukin-6 (IL-6), and tumor necrosis factor-alpha (TNF-α) are elevated, indicating an underlying metabolic imbalance [3]. CRP is a commonly used marker of systemic inflammation. Proinflammatory cytokines, such as IL-6 and TNF-α, are involved in immune activation and are closely associated with endothelial dysfunction and increased cardiovascular risk in patients with OSA. Each cycle of hypoxia and reoxygenation triggers immune signaling changes. Such fluctuations cause activation of the sympathetic nervous system along with endothelial impairment. Over time, these processes lead to higher risks for hypertension, diabetes mellitus, and atherosclerosis [4,5]. Given the role of chronic systemic inflammation in the development of cardiovascular disease, metabolic syndrome, and endothelial dysfunction, measurement of inflammatory biomarkers may provide insight into the systemic effects of OSA treatment beyond improvements in respiratory symptoms alone.

Although continuous positive airway pressure (CPAP) remains the gold standard for managing OSA, many individuals struggle to maintain consistent use over time [6]. As a result, alternatives such as oral appliance therapy (OAT), especially mandibular advancement devices (MAD), are now commonly considered when OSA severity ranges from mild to moderate or when CPAP is intolerable [7]. Instead of relying on pressurized airflow, these devices work mechanically by advancing the mandible, reducing obstruction risks during sleep. As disrupted breathing at night often contributes to systemic inflammation, interest has grown in studying whether OAT might influence underlying inflammatory markers linked to cardiac and metabolic health [8,9]. 

OAT effects on systemic inflammatory biomarkers have been studied in several clinical trials. However, the current evidence is limited due to small sample sizes, heterogeneous study design, differences in treatment duration and variability of biomarkers assessed, and their results are inconsistent. Randomized trials indicate no notable changes in major markers like IL-6, TNF-α, and CRP during short-term follow-up periods, especially among individuals with OSA and hypertension [10,11]. Similarly, certain intervention-based reports detect almost no alterations in pro-inflammatory cytokines after limited use of OAT [12], whereas isolated cases note changes in anti-inflammatory markers like IL-10 [13]. Literature has highlighted the variability in outcomes, noting that though changes may occur, the magnitude and consistency of these effects are uncertain [14].

When compared to OAT, treatments like CPAP and surgery often show significant reductions in markers of systemic inflammation, according to large-scale studies [15,16]. Sleep surgery correlates with significant decline in CRP, IL-6, TNF-α, along with other cardiometabolic markers, suggesting that inflammatory processes may reverse with effective OSA management [17]. However, results from CPAP are quite inconsistent. Some patient populations exhibit only mild or inconsistent changes in the inflammatory markers, pointing to a complex interplay between therapy and immune response [18]. Factors such as treatment time, initial severity of the condition, and treatment adherence appear to influence the outcomes across cases.

Though attention has grown, research into how OAT affects systemic inflammatory markers remains limited, inconsistent, and fragmented. Study methods differ greatly in terms of design of the study, number of samples, length of follow-up, types of oral appliances used, and the biomarkers studied. The devices range across designs, each possibly influencing outcomes differently. In addition, measured biomarkers are not uniform across trials, complicating comparisons. Many reports rely on small sample sizes, weakening statistical strength. Standardized assessment tools are rarely used [12,13]. As a result, firm interpretations become difficult. To date, there is a paucity of systematic reviews and meta-analyses that specifically evaluate the available literature on pre- and post-treatment changes in systemic inflammatory biomarkers with OAT in OSA patients. Given increasing clinical use of such therapies, this absence marks a gap in the research. Therefore, a comprehensive systematic review and meta-analysis is justified to synthesise the existing evidence, evaluate the consistency of current findings and identify the gaps that need to be filled in future clinical studies.

OAT was hypothesised to be associated with a decrease in systemic inflammatory biomarkers in patients with OSA, reflecting potential improvement in systemic inflammatory status. Accordingly, the aim of the present systematic review and meta-analysis was to provide a thorough assessment and summary of the available evidence on pre- and post-treatment changes in systemic inflammatory biomarkers following OAT in patients with OSA.

## Review

Materials and methods

Eligibility Criteria

This systematic review and meta-analysis followed the Preferred Reporting Items for Systematic Reviews and Meta-Analyses (PRISMA) guidance [19]. The present research was registered in PROSPERO with the ID CRD420261340212. The conduct of this systematic review and meta-analysis did not involve any deviations or amendments to the registered PROSPERO protocol. Eligibility criteria were defined by the Population, Intervention, Comparison, Outcome, and Study Design (PICOS) structure. Adult individuals with OSA receiving oral appliances, specifically MADs, were central to eligibility. Only those studies measuring changes in systemic inflammatory markers like CRP, interleukins, TNF-α, or other cytokines before and after treatment were qualified. Trials that were randomized controlled and observational designs, whether prospective or retrospective, met consideration standards. Excluded entirely were research on children, animals, case reports/case series, narrative reviews, conference papers, and investigations without marker data.

Information Sources

A comprehensive search of published research was conducted in several digital platforms - PubMed/MEDLINE, Scopus, Web of Science, and the Cochrane Library - with coverage extending from each database's inception to its latest update. Beyond these, grey literature sources like Google Scholar entered the process; further findings emerged through careful review of citations within selected papers.

Search Strategy

A comprehensive literature search was performed in PubMed/MEDLINE, Embase, Scopus, Web of Science, Cochrane Library and Google Scholar from inception until June 2026. We searched using a combination of medical subject headings (MeSH) and free-text keywords relating to obstructive sleep apnea, oral appliance therapy, mandibular advancement device, mandibular advancement splint, oral appliance, systemic inflammation, C-reactive protein, interleukin, tumour necrosis factor-alpha, and cytokines. The search terms were combined with Boolean operators (“AND” and “OR”). Searches were restricted (filters) to English-language articles. The reference lists of the included studies were also searched for additional eligible studies.

Selection Process

The selection process occurred in two stages. At first, two reviewers separately reviewed titles and abstracts of every record found, aiming to identify research that might qualify. Following that, those selected moved forward and their full texts were examined against set criteria for inclusion. When opinions differed, discussion happened to resolve it or a third reviewer offered insight. A PRISMA flow diagram traced each step taken during this filtering.

Data Collection Process

Two reviewers did the data extraction independently. Information gathered covered characteristics like author, year of publication, study design, number of participants, severity of OSA during sleep, type of OAT studied, along with follow-up period. Where differences appeared between findings, those involved were discussed until alignment occurred. Toward the end, consistency emerged after discussion addressed mismatches.

Data Items

Included data items were levels of inflammatory markers before and after treatment - CRP, interleukins, and TNF-α with accompanying measures of variability like standard deviations and confidence intervals. Participant characteristics covered characteristics such as age, gender, and body mass index. Therapy details appeared when recorded: treatment duration and adherence. These formed the secondary set of reported elements.

Risk of Bias Assessment

Assessment of risk of bias of included studies was performed by two reviewers independently. Where trials were randomized, evaluation relied upon the Cochrane instrument (ROB2) for detecting bias potential [20]. Risk Of Bias In Non-randomized Studies - of Interventions (ROBINS-I) was used for non-randomized studies [21]. Observational designs received scrutiny through the Newcastle-Ottawa Scale (NOS) instead [22]. Classification into low, moderate, or high risk of bias followed predefined benchmarks. Joanna Briggs Institute (JBI) was used for single-arm experimental studies [23]. 

Effect Measures

Continuous outcomes were synthesised using mean differences (MDs) with corresponding 95% confidence intervals (CIs). Mean differences were used because all the included studies reported inflammatory biomarker concentrations on the same measurement scale and in the same units and therefore no standardisation was needed. All meta-analyses were performed with the random-effects model. To maintain consistency across values, statistical conversions were done whenever necessary. 

Synthesis Methods

Statistical analyses were performed using Jamovi 2.7.18. To allow for differences across investigations, a random-effects model (REML) was used [24]. The degree of statistical inconsistency drew assessment through both I-squared values and Chi-square tests according to Cochrane recommendations [25]. Subgroup and sensitivity analyses were planned but not performed, as the limited number of eligible studies did not allow meaningful analyses. Evaluation adjustments followed whenever conditions permitted exploration of result stability. The meta-analysis only included studies that had enough quantitative data to calculate effect size. No imputations of missing outcome data were made.

Reporting Bias Assessment

If sufficient studies were available, funnel plots underwent visual inspection [24,25]. Examination via Egger’s regression technique followed to assess publication bias [26].

Certainty Assessment

The Grading of Recommendations Assessment, Development and Evaluation (GRADE) approach was used to assess the certainty assessment [27]. Strength levels were categorized as high, moderate, low, or very low. Patterns across data shaped these ratings, influenced by how broad the scope appeared and whether small samples played a role. Confidence shifted when gaps grew more pronounced.

Results

Selected Studies

**Figure 1:**
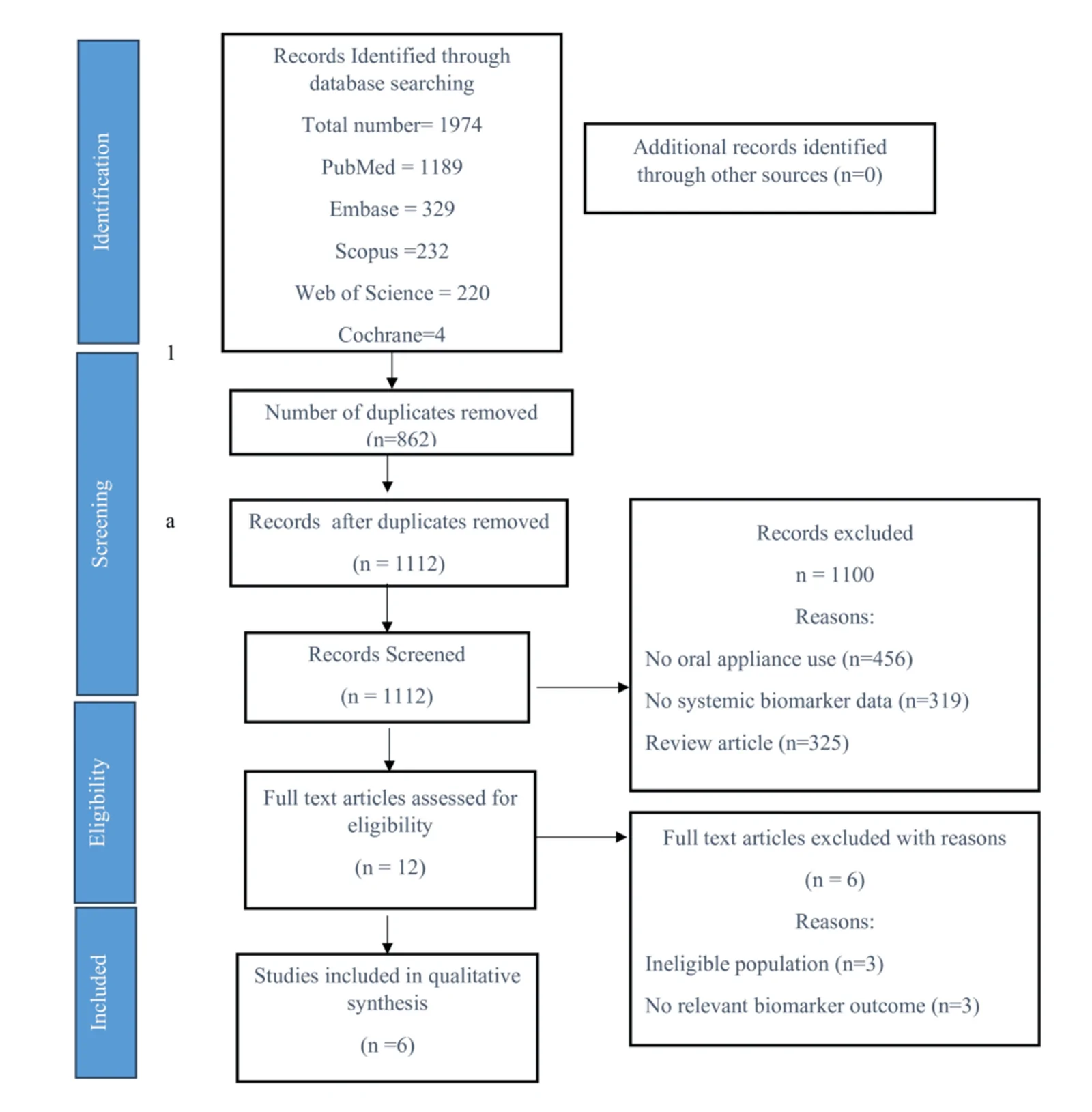
Preferred Reporting Items for Systematic Reviews and Meta-Analyses (PRISMA) 2020 Flow Diagram of Study Selection

The process of selecting studies is shown in Figure 1 according to PRISMA guidelines. Electronic database searches yielded a total of 1,974 records, including 1,189 from PubMed, 329 from Embase, 232 from Scopus, 220 from Web of Science and four from Cochrane Library. Other sources did not reveal any additional records. We removed 862 duplicate records and 1,112 records were left for title and abstract screening. Of these, 1,100 records were excluded as they did not meet the eligibility criteria, mainly due to absence of OAT interventions (n=456), lack of systemic inflammatory biomarker data (n=319), or were review articles (n=325). The remaining 12 articles were reviewed in full text for eligibility. Subsequently, six studies were excluded; three studies had ineligible populations and three studies did not report relevant biomarker outcomes. Finally, six studies met the inclusion criteria and were included in the qualitative synthesis and meta-analysis (Figure 1).

Characteristics of Included Studies

From the six analyzed investigations [12,13,28-31], involving 290 individuals experiencing different levels of OSA, the present review examined how oral appliances influence systemic inflammatory markers (Table 1). Study frameworks covered randomized trials alongside non-randomized comparisons and observational studies; intervention duration ranged from two to six months. Among measured indicators, emphasis fell on CRP, specific interleukins (IL-1β, IL-6, IL-10), and TNF-α, with some studies additionally evaluating oxidative stress markers and hemostatic parameters [13,28]. In general, the application of OAT showed significant reductions in breathing interruptions during sleep along with improved oxygen saturation, seen uniformly throughout each study, pointing toward reliable therapeutic effects for OSA [12,13,28-31]. Still, changes in systemic inflammation markers showed inconsistency across trials. Few studies, including work by Niżankowska-Jędrzejczyk et al. [28] and Fernandez-Julian et al. [29], demonstrated significant reductions in markers of inflammation, especially IL-1β and TNF-α, as well as certain coagulation-related biomarkers, which may indicate that OAT influences inflammation pathways.

**Table 1:** Characteristics of studies assessing the effect of oral appliance therapy on systemic inflammatory biomarkers in patients with obstructive sleep apnea. OSA: obstructive sleep apnoea; AHI: Apnea-Hypopnea Index; CRP: C-reactive protein; hs-CRP: high-sensitivity C-reactive protein; IL: interleukin; TNF-α: tumor necrosis factor-alpha; WBC: white blood cell count; PAI-1: plasminogen activator inhibitor-1; TAFIa: thrombin-activatable fibrinolysis inhibitor (activated form); TBARS: thiobarbituric acid reactive substances; AOPP: advanced oxidation protein products; NO: nitric oxide; ODI: Oxygen Desaturation Index; QoL: quality of life

Author (Year)	Study Design	Sample Size	Population Characteristics	Type of Appliance	Duration	Biomarkers Assessed	Key Findings
Niżankowska-Jędrzejczyk et al., 2014 [28]	Parallel controlled trial	22 OSA + 16 controls	Mild-moderate OSA; matched controls	Mandibular Advancement Splint (MAS)	3 & 6 months	CRP, IL-1β, IL-6, IL-10, fibrinogen, D-dimer, TAFIa, PAI-1	Significant improvement in IL-1β, D-dimer, TAFIa, and clot lysis time; inflammatory and hemostatic profiles improved to near control levels
Fernandez-Julian et al., 2017 [29]	Case-control study	30 treated + 10 control	Moderate-severe OSA	Mandibular Advancement Device (MAD)	6 months	CRP, IL-1β, IL-6, TNF-α	Significant reductions in IL-1β and TNF-α; improvement in CRP and sleep parameters; better QoL outcomes
Hedberg et al., 2020 [12]	Randomized controlled trial	71 patients	OSA with hypertension	Active vs passive oral appliance	3 months	hs-CRP, IL-6, IL-10, TNF-α, WBC	No significant changes in inflammatory biomarkers despite improvement in AHI
Peres et al., 2023 [13]	Quasi-experimental study	9 elderly patients	Elderly OSA patients	Intraoral appliance (IOA)	60 days	IL-6, TNF-α, IL-10, TBARS, AOPP, NO	Significant reduction in IL-10, oxidative stress markers (TBARS, AOPP); improvement in AHI and ODI
								Recoquillon et al., 2018 [30]	Randomized controlled trial	109 patients (55 MAD, 54 sham)	Severe OSA	MAD vs sham device	2 months	CRP, IL-6, TNF-α, leptin, adiponectin	No significant inter-group differences in inflammatory biomarkers despite AHI reduction
Yalamanchali et al., 2014 [31]	Retrospective study	49 patients	Mild-severe OSA	Custom MAD (TAP 3)	≥30 days	CRP	Significant reduction in CRP (2.5 → 1.9 mg/dL); AHI significantly reduced

Clear patterns emerge from rigorous trials: studies by Hedberg et al. [12] and Recoquillon et al. [30] revealed no major changes in systemic inflammatory markers, even though the respiratory parameters improved markedly, suggesting that short-term OAT might not consistently improve the markers of systemic inflammation. Still, reductions in CRP noted by Yalamanchali et al. [31], together with changes in oxidative indicators seen by Peres et al. [13], limit the generalizability of the results as they derive from small sample size or observational study design. Differences across investigations in sample size, trial length, methods used, and systemic inflammatory markers assessed contributed to inconsistency among findings. Collectively, the findings suggest that even though OAT effectively improves clinical and polysomnographic outcomes in OSA patients, its effect on systemic inflammatory biomarkers remains inconclusive, with potential benefits appearing more evident in longer-duration or specific patient subgroups.

Results of Meta-Analysis

**Figure 2:**
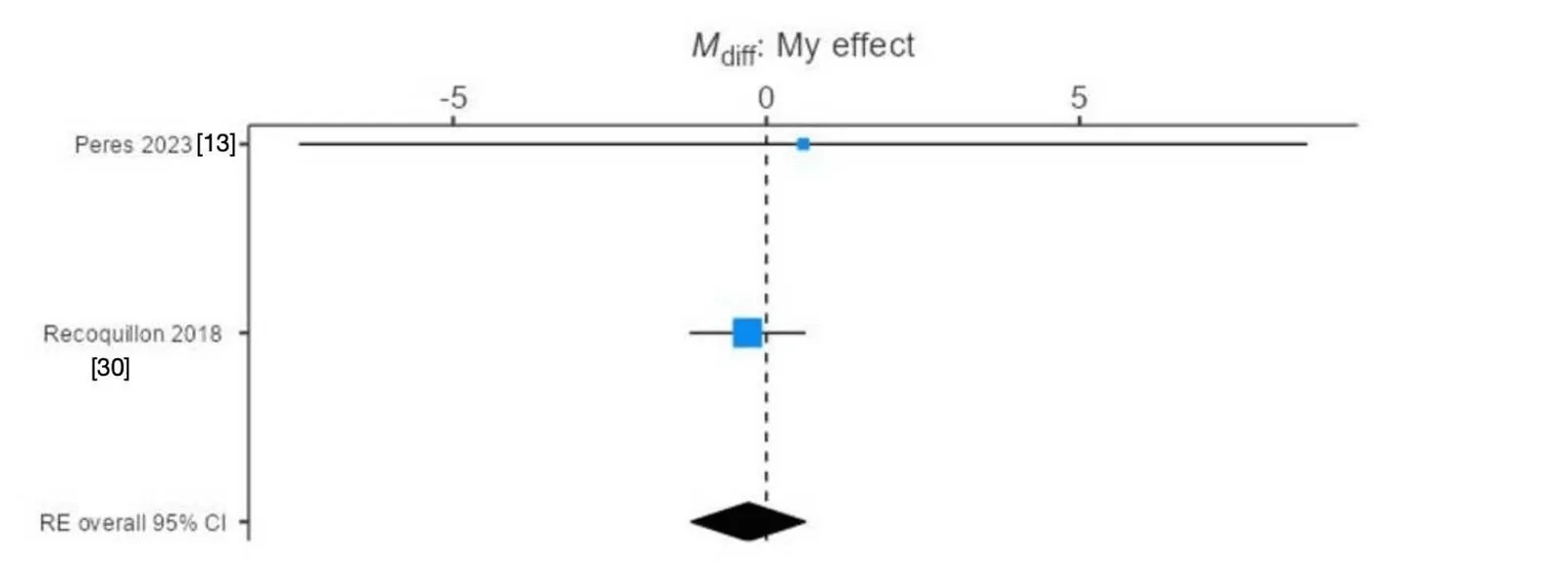
Forest plot of the effect of oral appliance therapy on TNF-α levels in patients with obstructive sleep apnea. Squares represent the study-specific effect estimates, the diamond represents the pooled effect size, and 95% confidence intervals (CI). RE: random-effects.

**Figure 3:**
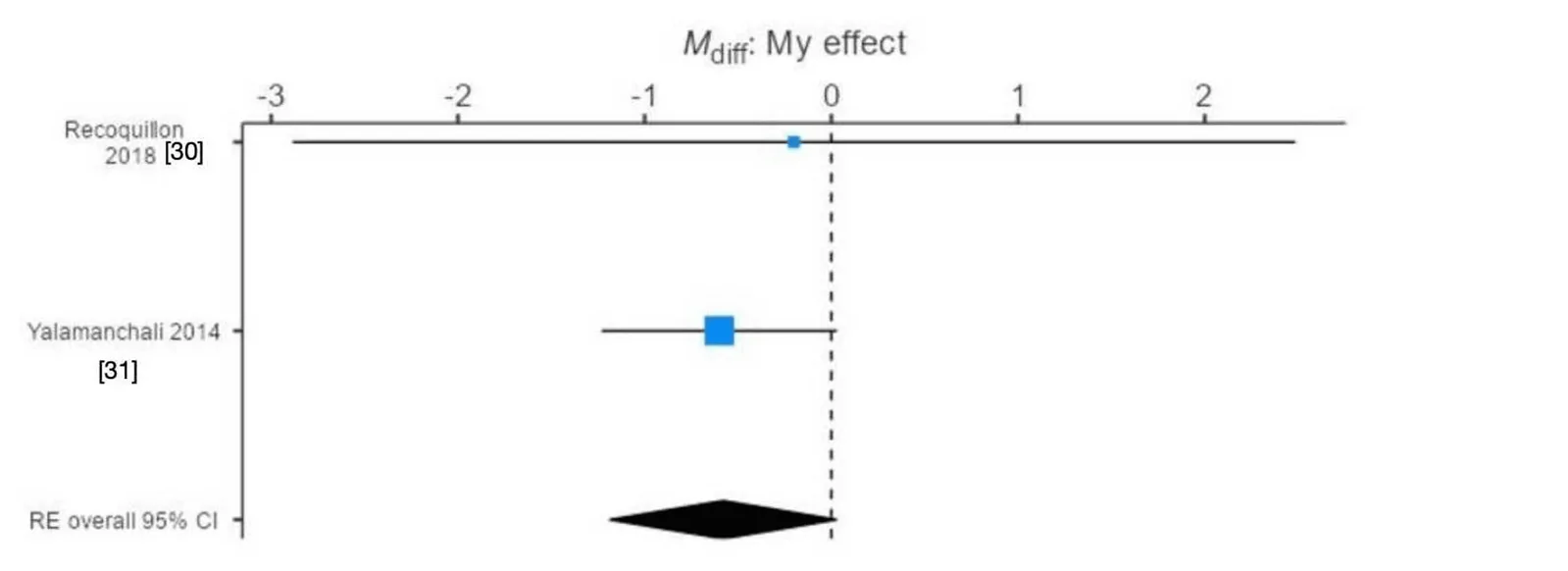
Forest plot of the effect of oral appliance therapy on CRP levels in obstructive sleep apnea patients. Squares represent study specific effect estimates and diamond represents pooled effect size with 95% confidence intervals (CI). RE: random-effects.

**Figure 4:**
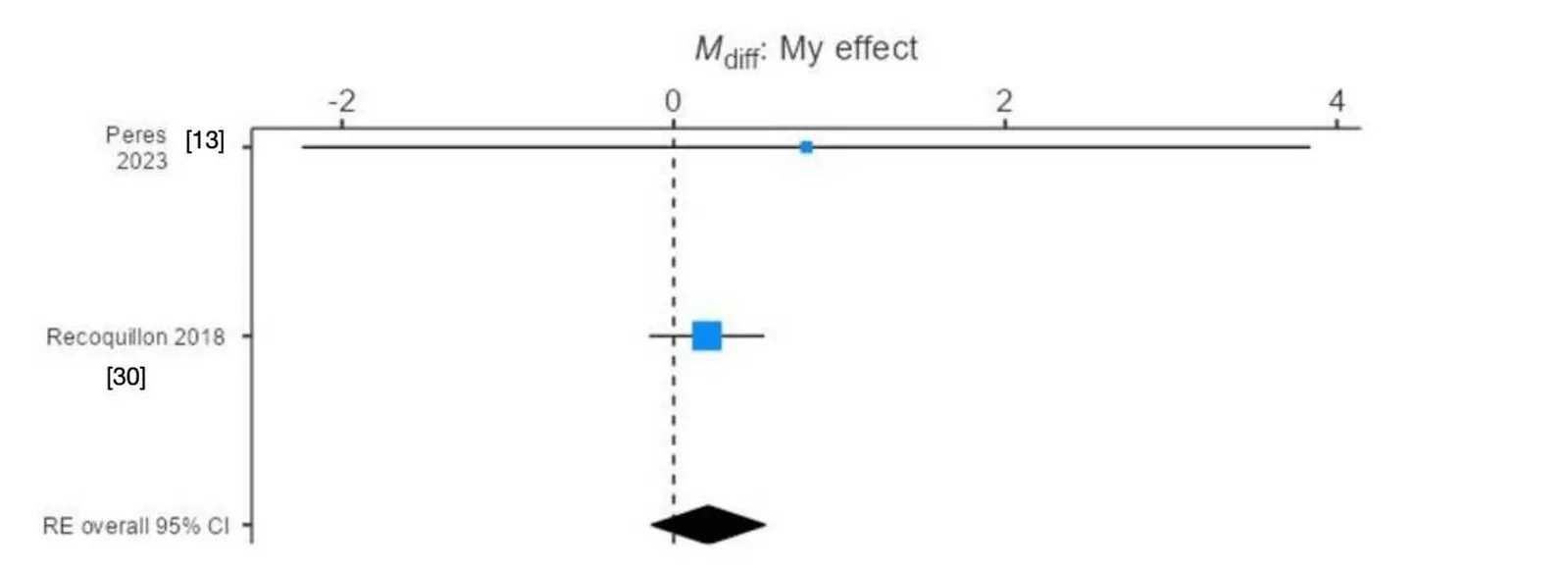
Forest plot of the effect of oral appliance therapy on IL-6 levels in patients with obstructive sleep apnea. Squares represent the study-specific effect estimates and the diamond the pooled effect size with its 95% confidence interval (CI). RE: random-effects.

The quantitative synthesis was performed for biomarkers reported in at least two studies, allowing inclusion of two studies per meta-analysis (Table 2, Figures 2-4). TNF-α levels reduced slightly with oral devices, though the change was not statistically significant (difference: -0.287; 95% CI: -1.20 to 0.622; p = 0.536), with consistency across studies (I² = 0%). CRP showed a reduction (difference: -0.579; 95% CI: -1.18 to 0.0261); however, the change was not significant (p = 0.061). On the other hand, IL-6 was increased non-significantly (difference: 0.209; 95% CI: -0.132 to 0.550; p = 0.230) (Table 3). Across these analyses, variation stayed minimal (I² = 0%) even if groups differed in size (Table 4). Although only two studies were available for each quantitative synthesis, the pooling was considered appropriate since the studies evaluated similar interventions and outcomes, estimating the direction and magnitude of effects while taking into account the limitations of a small evidence base [28,29]

**Table 2:** Effect estimates from individual studies included in meta-analysis of inflammatory biomarkers after oral appliance therapy RE: random-effects, LL: lower limit; UL: upper limit

Biomarker	Study	Mean Difference	95% CI (LL to UL)	RE Weight (%)	SE	p-value
TNF-α	Peres et al. 2023 [13]	0.59	-7.46 to 8.64	1.49	3.797	0.878
	Recoquillon et al. 2018 [30]	-0.300	-1.23 to 0.63	98.51	0.467	0.522
CRP	Recoquillon et al. 2018 [30]	-0.200	-2.88 to 2.48	5.2	1.354	0.883
	Yalamanchali et al. 2014 [31]	-0.600	-1.23 to 0.03	94.8	0.317	0.062
IL-6	Peres et al. 2023 [13]	0.8	-2.24 to 3.84	1.47	1.434	0.585

**Table 3:** Pooled effect estimates from random-effects meta-analyses of studies reporting quantitative pre- and post-treatment biomarker data after oral appliance therapy in patients with obstructive sleep apnea. LL: lower limit; UL: upper limit

Biomarker	Included studies	95% CI (LL to UL)	SE	k (studies)	p-value
TNF-α	Peres et al. 2023 [13]; Recoquillon et al. 2018 [30]	-1.20 to 0.62	0.464	2	0.536
						CRP	Recoquillon et al. 2018 [30]; Yalamanchali et al. 2014 [31]	-1.18 to 0.03	0.309	2	0.061
IL-6	Peres et al. 2023 [13]; Recoquillon et al. 2018 [30]	-0.13 to 0.55	0.174	2	0.23						

**Table 4:** Heterogeneity statistics for pooled analyses of inflammatory markers after oral appliance therapy.

Biomarker	Included studies	I² (%)	τ²	H²	Interpretation
TNF-α	Peres et al. 2023 [13]; Recoquillon et al. 2018 [30]	0	0	1	No heterogeneity
						CRP	Recoquillon et al. 2018 [30]; Yalamanchali et al. 2014 [31]	0	0	1	No heterogeneity
IL-6	Peres et al. 2023 [13]; Recoquillon et al. 2018 [30]	0	0	1	No heterogeneity						

This method improves understanding more than narrative synthesis, while also uncovering patterns invisible through qualitative analysis. Notably, when few studies exist, these evaluations still add value - revealing agreement across findings, shaping where future research should focus, and pointing toward the necessity of broader, well-designed trials needed to confirm how oral appliances affect markers of systemic inflammation.

Only biomarkers reported in two or more studies from which quantitative data could be extracted were meta-analysed. For TNF-α and IL-6, data from Peres et al. [13] and Recoquillon et al. [30] were included, and for CRP data from Recoquillon et al. [30] and Yalamanchali et al. [31] were included.

Publication Bias Assessment

Publication bias could not be assessed due to fewer than 10 studies per meta-analysis. Thus, we did not use funnel plot analysis and Egger’s regression test as these methods are unreliable when relying on a small number of studies.

Risk of Bias Assessment

**Figure 5:**
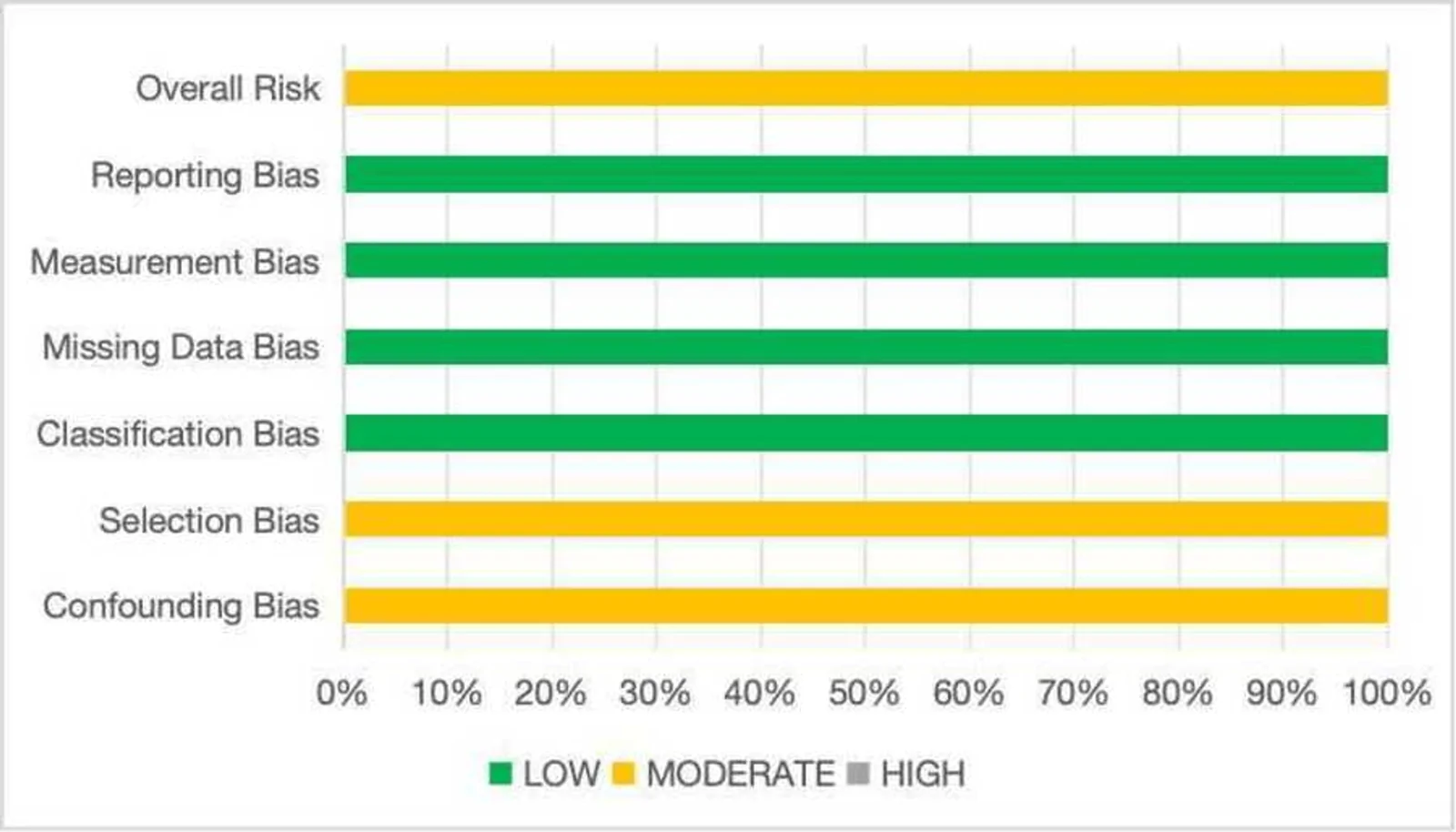
Risk of bias assessment using ROBINS-I tool ROBINS-I: Risk Of Bias In Non-randomized Studies - of Interventions [21]

**Figure 6:**
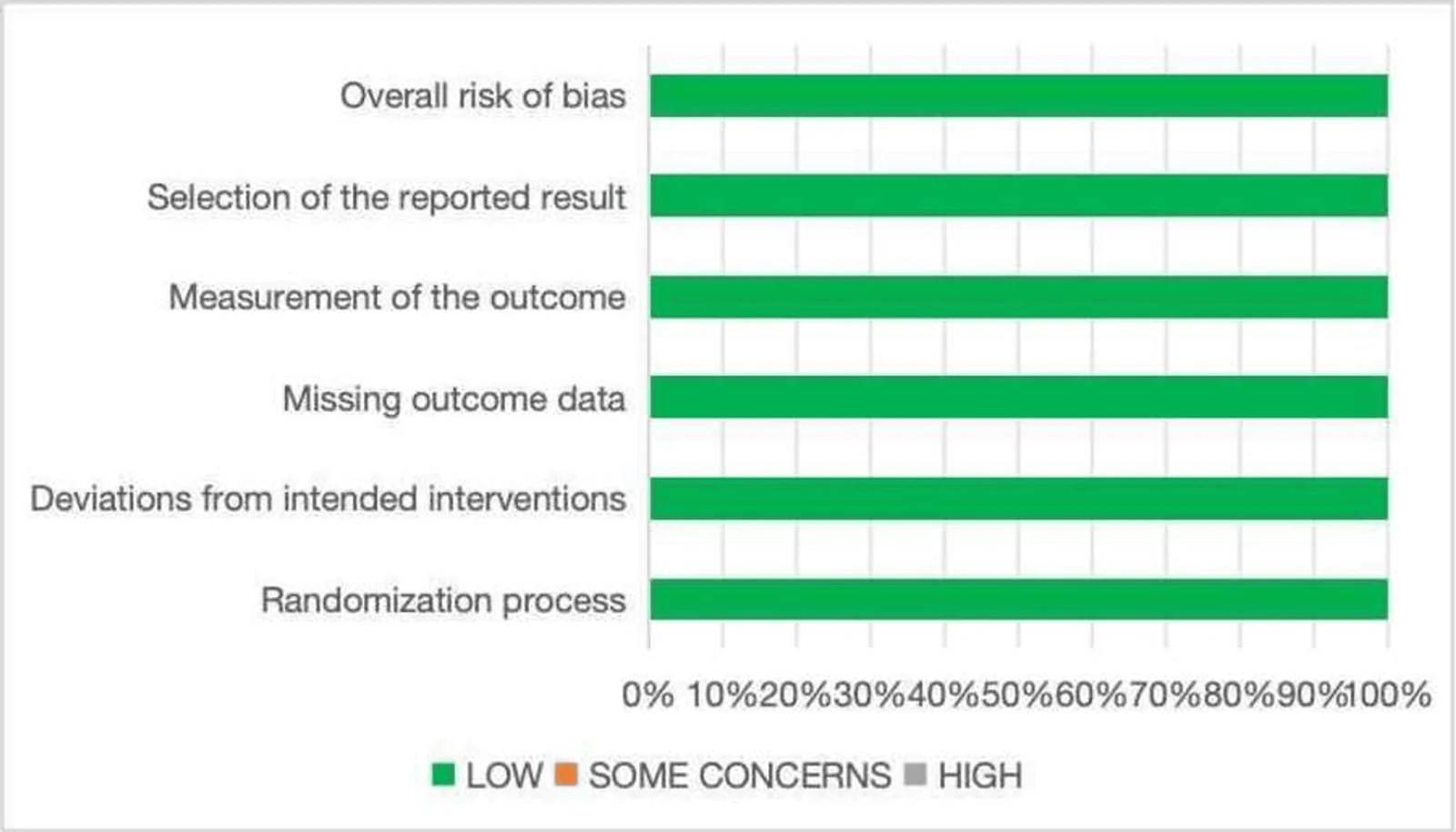
Risk of bias assessment using ROB-2 tool ROB-2: Revised Cochrane Risk of Bias tool for Randomized Trials [20]

**Figure 7:**
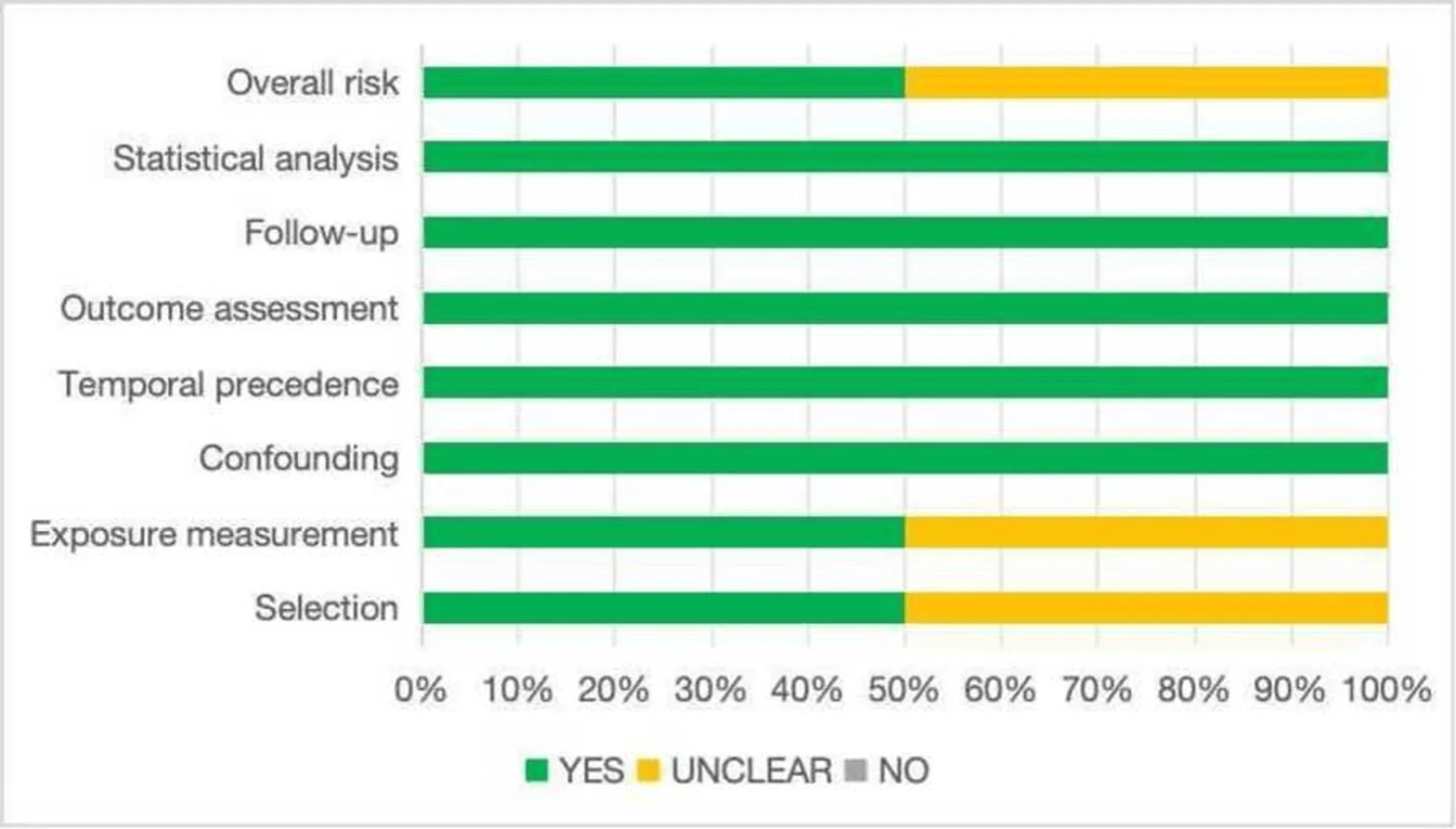
Risk of bias assessment using JBI tool JBI: Joanna Briggs Institute [23]

As the study designs of the included studies varied, distinct assessment tools were used to evaluate bias risks (Table 5, Figures 5-7). Low bias levels appeared consistently in randomised experiments [12,30], pointing to solid design practices. On the other hand, non-randomized comparative studies revealed moderate risk of bias, primarily due to confounding and participant selection [28]. When evaluated through JBI checklists and NOS, studies had moderate risk of bias owing to missing comparison groups and incomplete follow-up [13,29,31]. 

**Table 5:** Risk of bias assessment of included studies based on appraisal tools specific to study design. ROBINS-I: Risk Of Bias In Non-randomized Studies of Interventions [21]; NOS: Newcastle-Ottawa Scale [22]; RoB 2: Cochrane Risk of Bias Tool for Randomized Trials (Version 2) [20]; JBI: Joanna Briggs Institute [23]; RCT: randomized controlled trial

Author (Year)	Study Design	Tool Used	Key Bias Concerns	Overall Risk of Bias
Niżankowska-Jędrzejczyk et al. 2014 [28]	Non-randomized controlled	ROBINS-I	Potential confounding; non-random allocation	Moderate
Fernandez-Julian et al. 2017 [29]	Case-control	NOS	Selection bias; confounding	Moderate
					Hedberg et al. 2020 [12]	RCT	RoB 2	No major concerns across domains	Low
Peres et al. 2023 [13]	Single-arm quasi-experimental	JBI	Small sample size	Moderate
Recoquillon et al. 2018 [30]	RCT	RoB 2	Well-conducted randomization and outcome assessment	Low
Yalamanchali et al. 2014 [31]	Single-arm quasi-experimental	JBI	Retrospective design; selection bias	Moderate

Certainty Assessment

We assessed the certainty of the evidence for each pooled outcome using the GRADE approach. A Summary of Findings table is added (Table 6) to give an overview of the certainty ratings and reasons for downgrading. Overall, the certainty of evidence was low to moderate, primarily because of the risk of bias, imprecision from small sample size and limited number of included studies, and inconsistency in study characteristics.

**Table 6:** Certainty assessment of included studies using GRADE method GRADE: Grading of Recommendations Assessment, Development and Evaluation [27]

Outcome	No. of studies	Certainty (GRADE)	Reason(s) for downgrading
TNF-α	2	Low	Risk of bias, imprecision (small sample size, only two studies)
CRP	2	Low	Risk of bias, imprecision
IL-6	2	Low	Risk of bias, imprecision

Discussion

Principle Findings

This systematic review and meta-analysis examined how OAT influences systemic inflammatory markers in people who have OSA. Results show that although such treatment reliably enhances both clinical and polysomnographic outcomes like apnea frequency and blood oxygen levels, the impact on inflammatory marker stays unclear [12,13,28-31]. Pooled data revealed no significant changes seen in key markers including TNF-α, CRP, or IL-6, even if CRP appeared to have a slight reduction overall. The present meta-analysis did not find statistically significant change in TNF-α (pooled mean difference [MD] = -0.287; 95% CI: -1.20 to 0.62; p = 0.536), CRP (MD = -0.579; 95% CI: -1.18 to 0.03; p = 0.061), or IL-6 (MD = 0.209; 95% CI: -0.13 to 0.55; p = 0.230) after OAT. Overall, these findings are consistent with the randomised trial by Hedberg et al. [12] who found no significant changes in hs-CRP, IL-6, IL-10, TNF-α or white blood cell count after three months of OAT despite significant improvements in the Apnea-Hypopnea Index. Similarly, Recoquillon et al. [30] did not find any significant differences between the MAD and sham-device groups in CRP, IL-6, TNF-α, leptin or adiponectin levels after two months of treatment. Consistency among results points toward the direction of the effect; still, inclusion of only a few trials and limited participant counts weaken confidence in the outcome patterns.

Comparison With Previous Literature

The results of this systematic review and meta-analysis align closely with prior systematic reviews centered on CPAP use. The findings of the present review are somewhat different from the evidence reported for CPAP therapy. In a meta-analysis of randomised controlled trials, Zhu et al. [18] reported significant reductions in inflammatory markers such as CRP and IL-6 after CPAP treatment. In a similar vein, Friščić et al. [15] reported improvements in several inflammatory biomarkers after CPAP therapy. However, other studies such as Hunt et al. [32] and Zota et al. [33] revealed no or inconsistent biomarker changes, indicating that reduction of systemic inflammation may be dependent on treatment duration, baseline inflammatory status, and patient characteristics and not solely improvement in respiratory parameters. This inconsistency demonstrates the complex and multifactorial nature of systemic inflammation in OSA [34]. 

Literature shows that systematic reviews on OAT in OSA patients are rare unlike CPAP data. Available narrative reviews and observational studies show potential improvements in selected biomarkers [35,36]; however, this result comes from small or heterogeneous populations, similar to the results of the present review. In contrast, some individual studies have reported positive biomarker changes after OAT. A significant decrease in IL-1β and TNF-α was reported by Fernandez-Julian et al. [29] after six months of MAD therapy while Niżankowska-Jędrzejczyk et al. [28] found significant improvements in IL-1β, D-dimer, thrombin-activatable fibrinolysis inhibitor and clot lysis time after treatment. Yalamanchali et al. [31] observed that CRP levels decreased from 2.5 mg/dL to 1.9 mg/dL after MAD therapy. However, these positive findings were not consistently replicated in the studies and were often limited by small sample sizes and methodological heterogeneity.

Potential Biological Explanation

Importantly, the absence of statistically significant pooled effects in the present review does not necessarily mean absence of biological effect. Those individual studies that demonstrated reductions in CRP, IL-1β and TNF-α generally employed longer treatment durations of three to six months, whereas those with shorter follow-up periods often did not show significant changes in biomarkers. This finding suggests that a longer exposure to OAT may be necessary before the measurement of systemic anti-inflammatory effects.

Clinical Implications

This systematic review differs from previous literature in various aspects. Notably, attention centers on oral appliances instead of CPAP or surgeries, spotlighting a less examined domain. This review remains the first of its kind in studying the pre- and post-treatment changes of systemic inflammatory biomarkers in OSA patients under OAT. This review includes both qualitative and quantitative analyses which remained as a research gap in the previous literature on OAT in OSA patients. Methodologically, rigor arises via tailored bias assessments matched to study designs, alongside certainty ratings based on GRADE principles. In addition, the inclusion of various inflammatory biomarkers provides a clear understanding of the systemic effects of OAT when compared to previous reviews which focused on limited markers or single study designs.

This analysis adds to prior work through a concentrated review of data on how systemic inflammation changes after using OAT in people with OSA. Findings extracted from randomized and observational studies reveal inconsistent results among biological markers even when clinical signs and symptoms improve visibly. Notably, improved breathing function does not always align with detectable reductions in overall inflammation, thereby refining present knowledge on OSA pathophysiology and treatment outcomes. Such results hold clinical significance, indicating these appliances manage clinical symptoms well, though their ability to alter deeper inflammatory processes might be narrow or depend on extended use of the oral appliances.

Meta-analysis was considered appropriate because only two studies were included in each quantitative synthesis and the studies evaluated similar interventions (OAT), reported the same inflammatory biomarkers, and provided sufficient quantitative data to be pooled. The random-effects model enabled us to estimate the overall direction and magnitude of treatment effects, accounting for the variability between studies. However, the pooling of only two studies severely limits the statistical power and precision of the pooled estimates. Moreover, heterogeneity statistics such as I 2 should be interpreted with caution as they are not reliable when based on a very small number of studies. Thus, the pooled results should be regarded as exploratory, not conclusive, and the results need to be confirmed in larger well-designed studies with standardised outcome reporting.

Strengths

The strength of this systematic review lies in following PRISMA standards, ensuring structured reporting throughout. A thorough search spans several databases, built on a clear search strategy meant to cover all possible publications. Studies of varying designs like randomized/non-randomized are included, widening the scope. Reliability grows through bias appraisal tools shaped for specific study types, applied consistently. Statistical pooling adds precision by offering quantitative estimates of outcomes observed. Certainty in findings gains clarity via the GRADE framework, making judgments visible and grounded.

Limitations

Even with clear advantages, certain drawbacks need recognition. Although a few investigations contributed data on each marker, just two publications shaped overall results. This restricted the reliability of conclusions. Differences in methods, participant numbers, treatment periods, and measured biomarkers made aligning results difficult. Because some designs lacked control groups or relied on observation alone, they introduced potential bias, including confounding and selection bias. Measurement methods for biomarkers differed widely; standardization in reporting is missing, which added challenges when combining results. The limited count of selected studies made it impossible to evaluate publication bias through structured assessment. The low statistical power and the imprecision of the pooled estimates resulting from the inclusion of only two studies in each meta-analysis further decreased confidence in the stability and generalisability of the effect estimates.

Future Directions

Future research should include carefully structured randomized trials, using consistent methods to measure biological markers over extended periods. Research must maintain sufficient sample sizes to clarify how oral appliances affect the systemic condition. Investigations should group individuals by existing conditions, initial inflammation levels, and severity of OSA. Rather than grouping all patients together, sorting them this way could reveal the individuals who might benefit more from therapy. Beyond commonly studied biomarkers, newer biomarkers related to endothelial function and cellular stress deserve attention. Linking these markers with observable health changes would offer a complete picture. With time, such approaches may uncover various unknown patterns.

## Conclusions

Overall, this systematic review and meta-analysis showed that OAT can improve certain clinical outcomes in people who have OSA; still, whether it alters markers of systemic inflammation lacks clarity. Though data across studies fail to show significant changes in key markers like TNF-α, CRP, and IL-6 after treatment, there appears a slight reduction in CRP levels, possibly hinting at minimal influence on internal inflammation processes. Such outcomes underline how intricate associations may be present between disrupted breathing during sleep and systemic inflammatory activity, shaped by multiple patient-related and treatment-related factors. Despite being grounded in trials rated moderately reliable, conclusions draw caution due to narrow participant numbers, differing research frameworks, and methodological constraints. For this reason, existing data do not confirm any major influence of OAT on overall inflammation levels in OSA patients. To reach firm conclusions and clarify how such therapy affects systemic inflammatory pathways, more rigorous studies across larger groups will be needed, featuring consistent metrics alongside extended observation periods.
